# Central and Peripheral Nervous System Progenitors Derived from Human Pluripotent Stem Cells Reveal a Unique Temporal and Cell-Type Specific Expression of PMCAs

**DOI:** 10.3389/fcell.2018.00005

**Published:** 2018-02-06

**Authors:** Muwan Chen, Sofie H. Laursen, Mette Habekost, Camilla H. Knudsen, Susanne H. Buchholdt, Jinrong Huang, Fengping Xu, Xin Liu, Lars Bolund, Yonglun Luo, Poul Nissen, Fabia Febbraro, Mark Denham

**Affiliations:** ^1^Danish Research Institute of Translational Neuroscience, Nordic EMBL Partnership for Molecular Medicine, Aarhus University, Aarhus, Denmark; ^2^Department of Biomedicine, Aarhus University, Aarhus, Denmark; ^3^Beijing Genomics Institute, Shenzhen, China; ^4^Lars Bolund Institute of Regenerative Medicine, Beijing Genomics Institute-Qingdao, Qingdao, China; ^5^China National GeneBank, Beijing Genomics Institute, Shenzhen, China; ^6^Laboratory of Genomics and Molecular Biomedicine, Department of Biology, University of Copenhagen, Copenhagen, Denmark; ^7^Department of Molecular Biology and Genetics, Aarhus University, Aarhus, Denmark; ^8^Department of Health Science and Technology, Aalborg University, Aalborg, Denmark

**Keywords:** plasma membrane calcium ATPase, human pluripotent stem cells, neural stem cells, neuromesodermal progenitors, mesencephalic dopaminergic neurons

## Abstract

The P-type ATPases family consists of ion and lipid transporters. Their unique diversity in function and expression is critical for normal development. In this study we investigated human pluripotent stem cells (hPSC) and different neural progenitor states to characterize the expression of the plasma membrane calcium ATPases (PMCAs) during human neural development and in mature mesencephalic dopaminergic (mesDA) neurons. Our RNA sequencing data identified a dynamic change in ATPase expression correlating with the differentiation time of the neural progenitors, which was independent of the neuronal progenitor type. Expression of ATP2B1 and ATP2B4 were the most abundantly expressed, in accordance with their main role in Ca^2+^ regulation and we observed all of the PMCAs to have a subcellular punctate localization. Interestingly in hPSCs ATP2B1 and ATP2B3 were highly expressed in a cell cycle specific manner and ATP2B2 and ATP2B4 were highly expressed in a hPSC sub-population. In neural rosettes a strong apical PMCA expression was identified in the luminal region. Lastly, we confirmed all PMCAs to be expressed in mesDA neurons, however at varying levels. Our results reveal that PMCA expression dynamically changes during stem cell differentiation and highlights the diverging needs of cell populations to regulate and properly integrate Ca^2+^ changes, which can ultimately correspond to changes in specific stem cell transcription states.

## Introduction

Cytosolic Ca^2+^ is an abundant intracellular second messenger involved in many cellular processes. A diverse array of proteins are able to bind to Ca^2+^ each with a varying range in affinity, from which the calcium binding can alter the protein's shape and charge, resulting in a potential functional change and alteration in cellular signaling. As such, of particular importance are the Ca^2+^ P-type ATPases, the plasma membrane calcium ATPases (PMCAs) and the sarco/endoplasmic reticulum Ca2+ ATPase (SERCAs), which reside in different compartments of the cell and along with other Ca^2+^ transporting system contribute to the regulation of the intracellular Ca^2+^ concentration.

The PMCA's ability to extrude and regulate Ca^2+^ levels is critical for cellular function and in turn the precise regulation of PMCA expression is also essential. The C-terminal of the PMCA is the main regulatory site of its pump and contains a calmodulin (CaM) binding domain and phosphorylation sites (Penniston and Enyedi, 1998; Brini and Carafoli, 2011; Tidow et al., 2012). Increased intracellular Ca^2+^ results in CaM stimulating pump activity by bending the C-terminal regulatory domain away from the active site of the pump releasing it from the auto-inhibitory state (Brini and Carafoli, 2011; Tidow et al., 2012). Phosphorylation in the regulatory C-terminal is executed by both Protein Kinase A (PKA) and Protein Kinase C (PKC); PKA stimulate Ca^2+^ extrusion, whereas PKC inhibits (James et al., 1989; Enyedi et al., 1997). PKC conducts its inhibitory effect by phosphorylation of the CaM binding domain, creating a barrier for CaM interaction and hereby inhibiting CaM stimulation (Enyedi et al., 1997). PMCA regulation of Ca^2+^ is therefore crucial in fine-tuning the levels of Ca^2+^ in the cytoplasm with sequence variability, splice variants, and cell type specific expression seen between the different PMCA isoforms all having varying effects on the multitude of affected signaling pathways.

Regulation of signaling pathways by Ca^2+^ occurs at the earliest stages of development where it plays a critical role in fertilization and hPSC maintenance (Deguchi et al., 2000; Todorova et al., 2009). Furthermore, Ca^2+^ has been interconnected with several neural properties such as synaptic transmission and long-term potentiation and dysregulation of intracellular calcium due to altered PMCA expression has been shown to affect neural differentiation through their involvement in Fgf and Wnt signaling (Brini et al., 2014; Abdul-Wajid et al., 2015; Boczek et al., 2015). Different PMCA genes maintain different expression profiles. In humans ATP2B1 and ATP2B4 are expressed throughout the body, whereas the two other isoforms, ATP2B2 and ATP2B3, exhibit neural and muscular tissue specific expression (Stauffer et al., 1995). Mutations in ATP2B1 is embryonic lethal and mutations in ATP2B2-4 have been correlated with different neuronal deficits: hearing loss, congenital ataxias, and familial spastic paraplegia, respectively (Okunade et al., 2004; Ficarella et al., 2007; Zanni et al., 2012; Ho et al., 2015). These studies highlight the important role of the various PMCAs in Ca^2+^ handling across the diverse cell types, therefore identifying their cell type specific expression during development is crucial for understanding cell type specific Ca^2+^ regulation.

The precise localization and neural specific expression of PMCAs during the development of the human nervous system is poorly understood. To address this we used hPSCs to generated neural stem cells (NSCs) of the central and peripheral nervous system and investigated the main neural progenitor states for the presence of PMCAs using RNA sequencing (RNA-seq) and immunofluorescent labeling. Our results uncovered a dynamic change in ATPase expression that correlates directly with the stage of differentiation. Furthermore, PMCA expression was not only altered between stem cell states, but also in addition showed unique cell cycle specific changes. Lastly due to the importance of Ca^2+^ regulation in Parkinson's disease (PD) we differentiated the NSC further and generated mesencephalic dopaminergic (mesDA) neurons to characterize the presence of PMCA proteins (Schöndorf et al., 2014). These data have important implications for understanding the role of Ca^2+^ in development and potentially how disease states, which disrupted Ca^2+^ homeostasis, can result in global cellular dysfunction.

## Materials and methods

### Human pluripotent stem cell culture

H9 (WA-09, WiCell) and iPSC-CCD (reprogrammed from human Foreskin fibroblasts, ATCC) cell lines were cultured as previously described (Denham and Dottori, 2011). Briefly, hESCs and hPSCs were cultured on irradiated human foreskin fibroblasts (HFF) in KSR media consisting of DMEM/nutrient mixture F-12, supplemented with β-mercaptoethanol 0.1 mM, non-essential amino acids (NEAA) 1%, glutamine 2 mM, penicillin 25 U/ml, streptomycin 25 μg/ml, and knockout serum replacement 20% (all from Life Technologies), supplemented with FGF2 (10 ng/ml; Peprotech) and Activin A (10 ng/ml; R&D systems). All cells were cultured at 37°C 5% CO_2_. Colonies were mechanically dissected every 7 days and transferred to freshly prepared HFF. Media was changed every second day.

### Neural stem cell differentiation

hESCs or hPSCs were mechanically dissected into pieces ~0.5 mm in diameter and transferred to laminin-coated organ culture plates in N2B27 medium containing 1:1 mix of neurobasal medium with DMEM/F12 medium, supplemented with insulin/transferrin/selenium 1%, N2 1%, retinol-free B27 1%, glucose 0.3%, penicillin 25 U/ml, and streptomycin 25 μg/ml (all from Life Technologies) for 11 days. Cultures were grown on laminin for the first 4 days after which they were dissected into 0.5 mm pieces and cultured in suspension in low-attachment 96-well plates (Corning) in N2B27 medium. For neural epithelial progenitors (NEP) specification SB431542 (SB; 10 μM, Tocris) was added to the media and cells were collected at day 4. To further specify the cells to rostral neural stem cell (NSC), NEP were generated and then further cultured to day11 in the absence of SB and supplemented with FGF2 (20 ng/ml; Peprotech). For neuromesodermal progenitors (NMP) induction, NEP induction was performed as above and cultures were additionally supplemented with GSK3β inhibitor CHIR99021 (CHIR; 3 μM, Stemgent) from days 0 to 4. For neural crest stem cells (NCSCs) specification NMP induction was performed and from days 4 to 11 cultures were supplemented with FGF2 (20 ng/ml; Peprotech) and BMP2 (50 ng/ml; Peprotech). For caudal NSC specification NMP induction was performed and from days 4 to 11 cultures were supplemented with FGF2 (20 ng/ml; Peprotech).

### Mesencephalic dopaminergic neuron differentiation

Generation of mesDA neurons was achieved by modification of previous described protocols (Denham et al., 2012; Kirkeby et al., 2012). Briefly, from day 0 to day 9 cells were grown in N2B27 media with 10 μM SB431542, 0.7 μM CHIR99021, 0.1 μM LDN-193189 (Stemgent), and 400 nM SAG (Millipore), day 4 to day 9 basal media change to ½ N2B27. At day 9 to day 11, the media was changed to ½ N2B27 without small molecules. From Day 11 the cells were grown in Neurobasal media supplemented with B27 1%, Pen/Strep 25 U/mL, Glutamax 0.5%, 200 μM Ascorbic Acid (AA) (Sigma-Aldrich) and grown on culture plate coated with polyornithine, fibronectin, and laminin (all from Sigma). From day 14, 2.5 μM DAPT (Tocris bioscience) was added into the culture media. The media was changed every second day and samples were collected on day 45.

### Immunolabeling

Cell monolayers and neurospheres were fixed in 4% PFA for 20 min at 4°C and then washed briefly in PBS. Neurospheres were embedded in Tissue-Tek OCT compound (Labtek), cut at 10 μm on a cryostat, and sections were placed on superfrost slides. Sections or culture dishes were blocked for 1 h at room temperature (RT) in blocking solution. The following primary antibodies were used: goat anti-Sox10 (1:100, R&D systems), goat anti-Sox2 (1:100, R&D), mouse anti-Sox2 (1:100 R&D), mouse anti-Oct4 (1:100, Santa Cruz), mouse anti-Nanog (1:100, eBioscience), mouse anti-ßIII-tubulin (1:1,000, Millipore), goat anti-FOXA2/HNF3Beta (1:500, Santa Cruz), mouse anti- Tyrosine hydroxylase (TH, 1:2000, Millipore), rabbit anti-ATP2B1 (1:1,000, SWANT) (Stauffer et al., 1995, 1997), goat anti-ATP2B2 (1:200, S-18 sc-22073, Santa Cruz) (Sahly et al., 2012), rabbit anti-ATP2B3 (1:1000, SWANT) (Stauffer et al., 1995, 1997), and goat anti-ATP2B4 (1:200, Y-20 sc-22080, Santa Cruz) (Patel et al., 2013). Antibodies were diluted in blocking solution incubated on sections and cultures overnight at 4°C. Following three 10-min washes in PBT, the corresponding Alexa Fluor-647, Alexa Fluor-488 or Alexa Fluor-594 donkey secondary antibodies were applied for 1 h at RT (1:400, Jackson ImmunoResearch). Nuclei were counterstained with 49,6-diamidino-2-phenylindole (DAPI; 1 μg/ml, Sigma). Slides were mounted in PVA-DABCO for viewing under a fluorescent microscope (ZEISS ApoTome), and images captured using the ZEN software. Confocal microscopy was performed using a ZEISS LSM 780 Confocal Microscope. The images were reconstructed as an intensity projection over the Z-axis using ZEN software.

### RNA sequencing

RNA sequencing was performed in collaboration with BGI-Research, Shenzhen, China. Briefly, total RNA was first assessed with Agilent 2100 Bioanaylzer and treated with DNase I. Magnetic beads with Oligo dT were used to isolate mRNA. The mRNA was then fragmented into short fragments with fragmentation buffer and complement DNA (cDNA) was synthesized using the mRNA fragments as templates. Short fragments were purified with EB buffer for end reparation and single nucleotide A (adenine) addition. After that, the short fragments were linked to adapters. After agarose gel electrophoresis, the suitable fragments were selected for the PCR amplification as templates. During the QC steps, Agilent 2100 Bioanaylzer and ABI StepOnePlus Real-Time PCR System were used for quantification and qualification of the sample library. Finally, the library was sequenced using Ion proton platform. Raw data from the Ion proton was subjected to data QC. Raw reads were filtered into clean reads and aligned to the reference gene with TMAP to calculate distribution of reads on reference genes and perform coverage analysis. At the same time, the clean reads were aligned to ref genome (hg19) with TopHat for a series of subsequent analysis. Finally, gene expression level and differential expression analysis was performed.

### Statistical analysis

One-way ANOVAs and post-hoc analyses were performed for statistical analyses on RPKM values using GraphPad Prism 6. To generate Heatmaps, RPKM values were log2 transformed and mean centered and graphed using R studio with heatmap.2. Principal components were calculated by singular value decomposition of the centered and scaled data matrix using the prcomp function in R stats package.

## Results

### Profiling of ATPase expression in human embryonic stem cells and in early neural stem cell states by RNA sequencing

**Figure 1:**
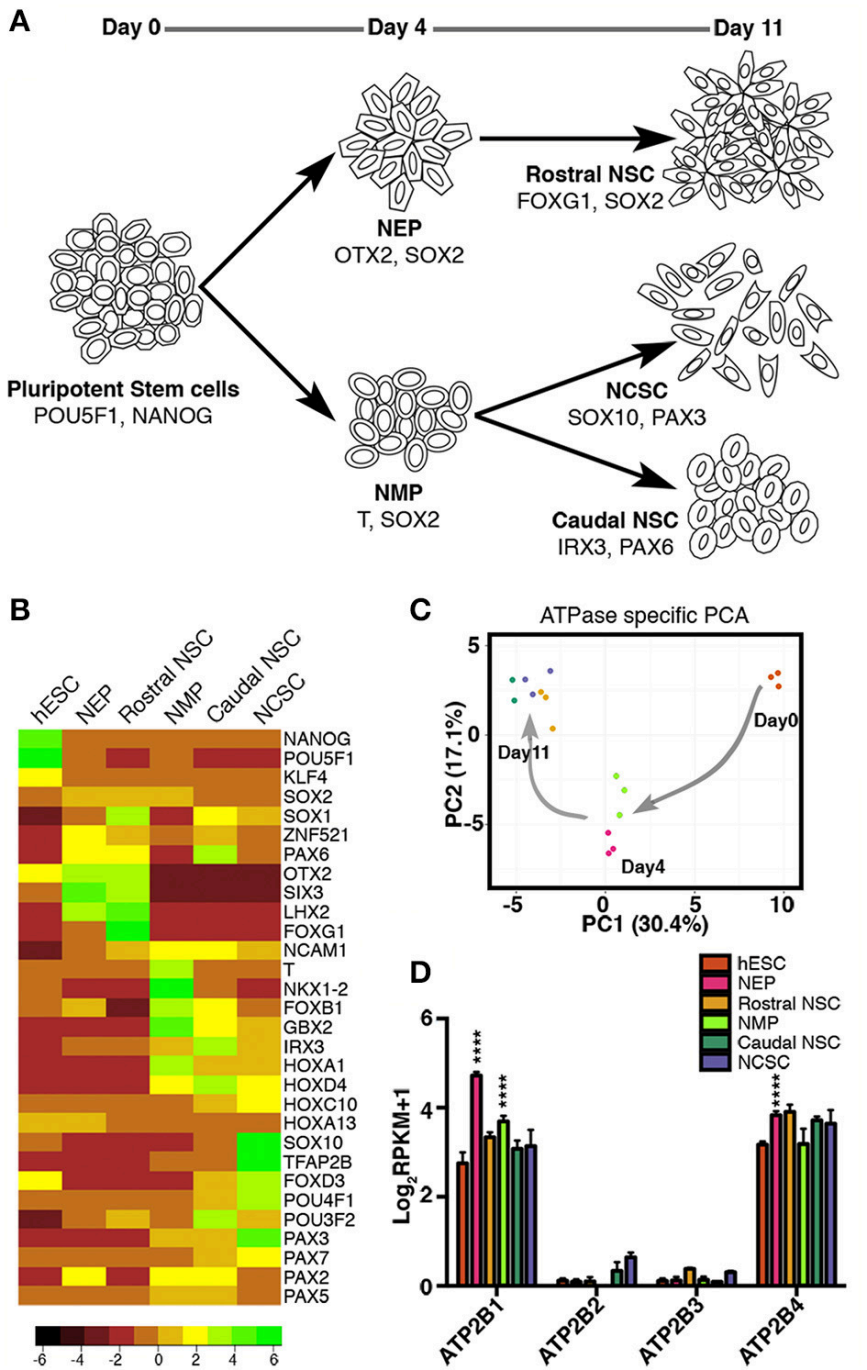
RNAseq profiling of ATPase expression in human embryonic stem cells and in early neural stem cell states. **(A)** Overview of protocol for deriving neural epithelial progenitors (NEP), and neuromesodermal progenitors (NMP) on day 4; rostral neural stem cells (NSC), neural crest stem cells (NCSCs) and caudal NSC on day 11. **(B)** Gene expression of known markers indicative of the various stem cell states. **(C)** Principal component analysis of expression of ATPases during early neural progenitor states. **(D)** RNAseq profiling of ATP2B1-4 expression (in RPKM values) in human embryonic stem cells and in early neural stem cell states (^****^p < 0.001). Not all significant differences are indicated. Legend shown in **(D)** also represents colored dots in **(C)**.

We first set out to characterize ATPase transcripts in hESCs and hESC-neural derivatives, including early NSCs of the central and peripheral nervous system. To generate CNS and PNS progenitors we implemented our previously described protocol (Denham et al., 2012, 2015). Day 4 NEPs along with the subsequent day 11 rostral NSCs were derived (Figure 1A). Additionally NMPs were generated and by further differentiating the day 4 NMPs in two distinct ways, caudal neural progenitors (day11; CNPs) and day 11 NCSCs were derived (Figure 1A). With these 6 distinct time points we set out to validate the gene expression of these populations by RNA-seq analysis (undifferentiated hESC and the five NSC states: NEP, NMP, rostral NSC, caudal NSC, and NCSC). Firstly we examined the gene expression of known markers indicative of the various stem cell states. As expected we identified *POU5F1* (previously known as *OCT4*) and *NANOG* as highly expressed in the undifferentiated hESCs and down-regulated in all of the neural populations: *SOX2* was maintained throughout all progenitor states (Figure 1B). Furthermore, day 4 NEP expressed transcripts indicative of anterior identity, such as *OTX2, SIX3*, and *LHX2*. Rostral NSCs expressed high levels of *SOX1, OTX2 FOXG1, SIX3*, and *LHX2*. NMPs as previously described are known to co-express *T* and *Sox2* (Tzouanacou et al., 2009; Gouti et al., 2014; Turner et al., 2014; Denham et al., 2015). In accordance with this we detected both high levels of *T* and *SOX2* in NMPs and we also detected *NKX1-2* and *FOXB1* (Figure 1B; Turner et al., 2014). Caudal neural markers were also detected such as *HOXA1* in the NMP state. Caudal NSCs expressed *IRX3* and *HOXD4*, additional *HOX* genes were also expressed with the most caudal representing the lumbar regions of the spinal cord (Figure 1B; Figure S1). NCSCs expressed the crest marker *SOX10, TFAP2B, FOXD3, PAX3*, and *POU4F1* (previously known as *BRN3A*).

To explore the variation in expression of ATPases during early neural progenitor states we performed a principal component analysis on all ATPases. Strikingly we observed that principal component 1 (PC1), which had the highest percentage of variance (30.4%) separated the stem cell states based on the time of differentiation, whereas the PC2 could separate only some of the stem cell states at each time point (Figure 1C). These results indicated that a dynamic change in ATPase expression occurs between the distinct progenitor states, progressively changing as the neural progenitors developed toward more committed progenitors.

Based on these results we further sought to examine the P-type ATPase PMCA members (*ATP2B1-4*; Figure 1D). *ATP2B1-4* were all examined between each of the stem cell states. *ATP2B1* and *ATP2B4* had higher amount of transcripts across all of the stem cell populations (Figure 1D). Interestingly the *ATP2B1* expression significantly increased (*p* < 0.0001) when differentiated from the pluripotent state to both NEP and NMP day4 progenitor states. In the NEP group *ATP2B4* expression was also significantly increased compared to the pluripotent state (*p* < 0.0001; Figure 1D). *ATP2B2* and *ATP2B3* were also detected, in all groups, except for in the NMP group where *ATP2B2* was undetected (Figure 1D).

### PMCA expression in human pluripotent stem cells

**Figure 2:**
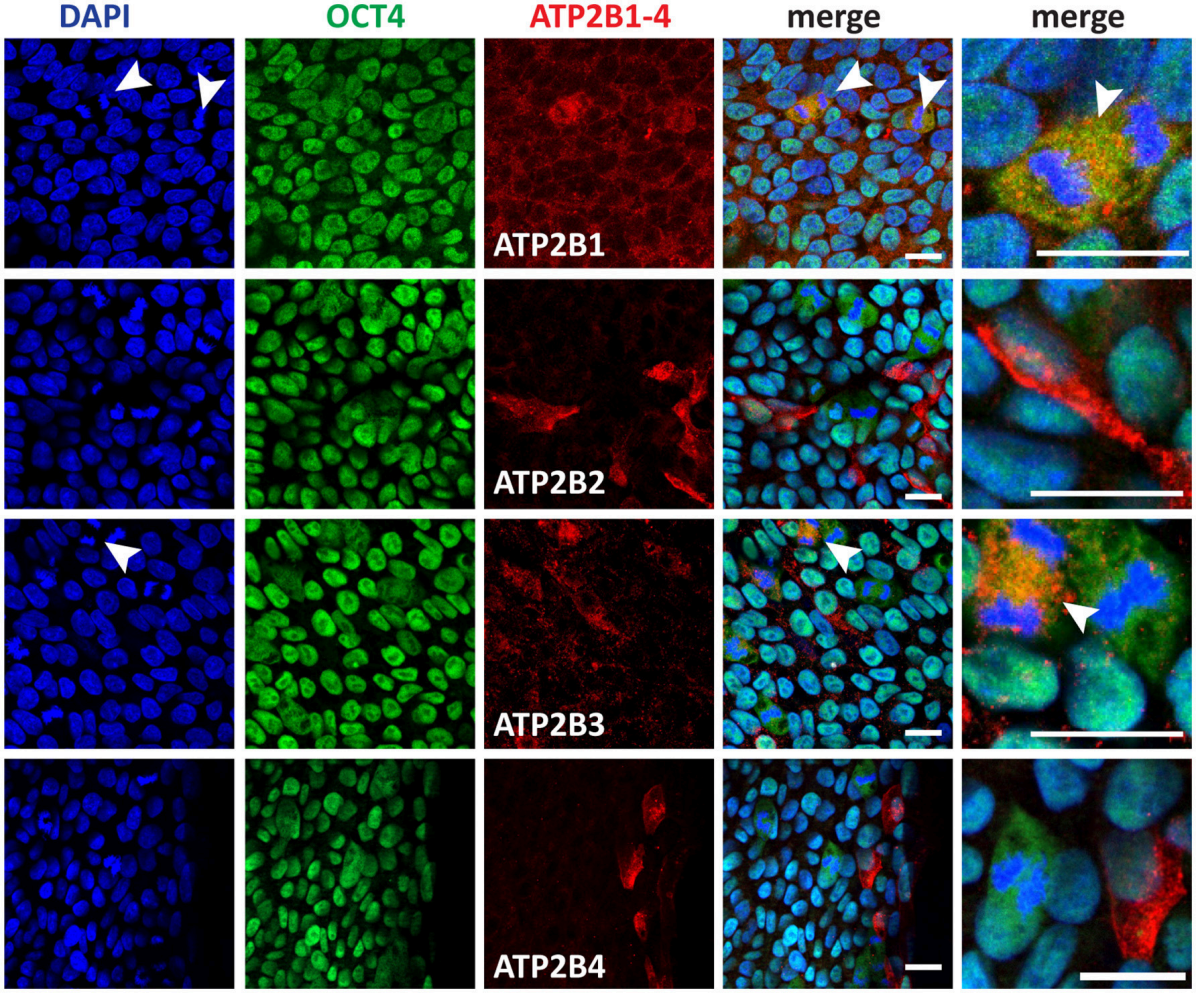
Immunocytochemistry analysis of ATP2B1-4 and POU5F1 expression in human pluripotent stem cells. Arrow heads point to the dividing cells. Scale bars = 20 μm.

We next investigated whether the expression of PMCAs, as indicated by RNAseq analysis (Figure 1D), were translated into detectable protein levels and to subsequently determine the cell subtype specific expression. ATP2B1-4 were all detected in pluripotent stem cells and showed a punctate localization pattern within the cells (Figure 2). ATP2B1 was ubiquitously expressed and interestingly vastly higher amounts were seen in dividing cells that also maintained expression of POU5F1 (Figure 2, arrow head). To further validate the pluripotent status of these cells NANOG expression was analyzed and, indeed, high ATP2B1 expressing cells also co-expressed NANOG (Figure S2).

In contrast, ATP2B2 expression was more restricted to a subpopulation of the hPSCs that could be identified also by an atypical morphology, consisting of a larger cytoplasm to nuclear ratio. Despite this morphology, POU5F1 and NANOG were both expressed in the ATP2B2 positive cells within the pluripotent culture. Notably however these cells were frequently located closer to the edges of the colony.

ATP2B3 showed a similar ubiquitous expression pattern to ATP2B1 and higher expression in dividing cells. However, interestingly, not all dividing cells expressed higher levels of ATP2B3, potentially representing a discrete stage during mitosis (Figure 2). Furthermore, ATP2B3 positive cells expressed POU5F1 and NANOG (Figure 2, Figure S2). Lastly we identified ATP2B4 positive cells that corresponded with a population close to the edge of the colony, with all positive cells having a large cytoplasm similar to ATP2B2 (Figure 2). Moreover, these cells also still expressed POU5F1 and NANOG.

Based on our analysis of hPSCs, all PMCAs were expressed in pluripotent, POU5F1 and NANOG positive, cells. The expression of PMCAs varied, however, with respect to cell morphology and cell cycle stage, with the most notable change being ATP2B1 and ATP2B3 expression being highest during cell division, a point at which cell differentiation can occur. Overall, precise regulation of Ca^2+^ by PMCAs could be required during mitosis in hPSCs. Furthermore, ATP2B2 and ATP2B4 were highly expressed in cells with morphologies indicative of a metastable differentiation state.

### PMCA expression in early neural stem cells

**Figure 3:**
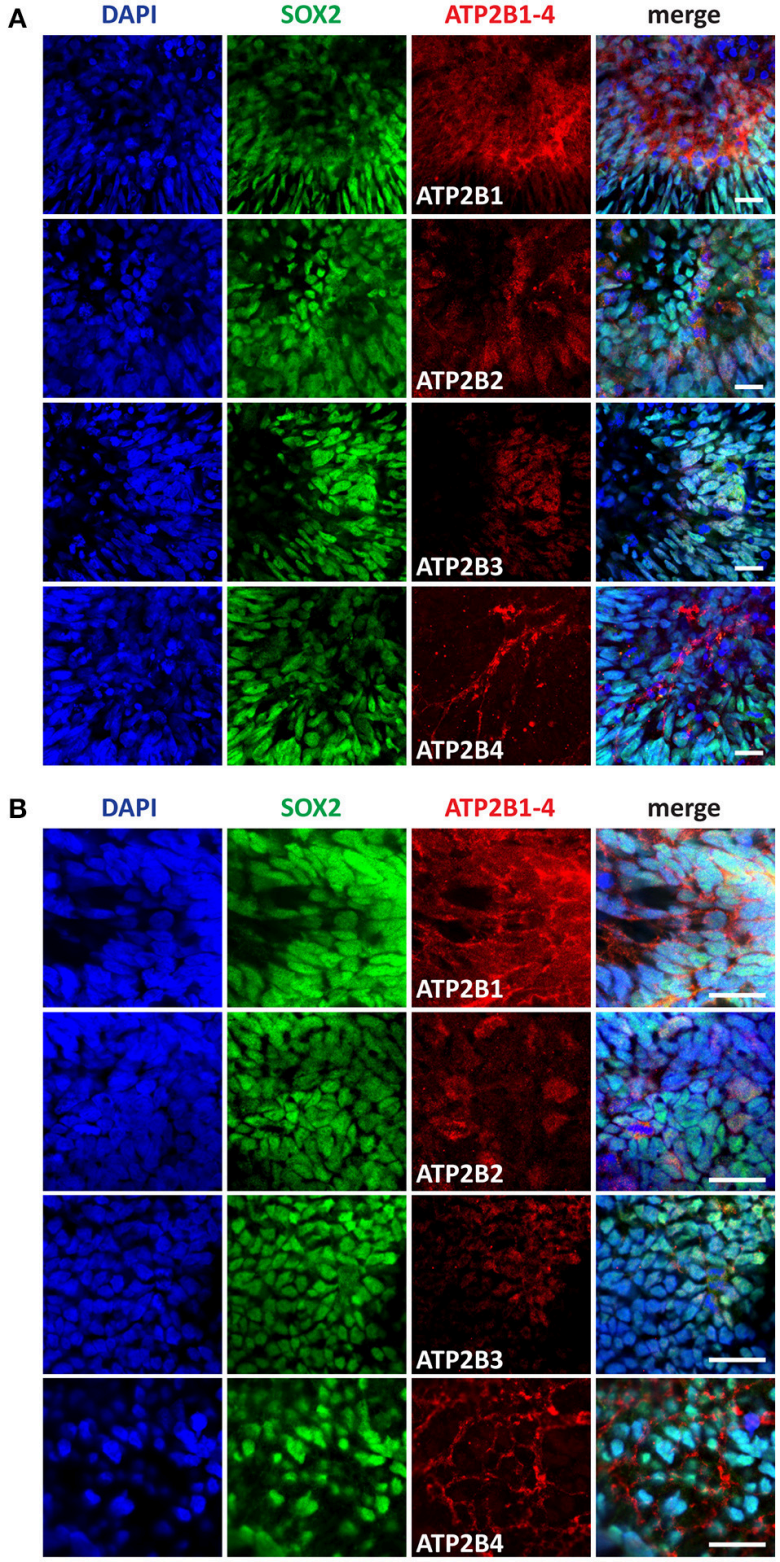
Immunocytochemistry analysis of ATP2B1-4 and SOX2 expression in **(A)** neural epithelial progenitors (NEP) and in **(B)** neuromesodermal progenitors (NMP) on day 4. Scale bars = 20 μm.

Differentiation of pluripotent stem cells toward a neuroectoderm fate coincides with transient increases in intracellular Ca^2+^. Based on our RNAseq data we identified a significant change in expression between pluripotent and day 4 time points for ATP2B1 (Figure 1D). Additionally, the higher expression of PMCAs in morphologically larger hPSCs and during cell division indicates a changing cellular state with varying requirements for Ca^2+^ regulation. We therefore further investigated the PMCA protein expression and localization during neural development. NEPs and NMPs were examined, which represent progenitors of the same differentiation time point but with vastly different potential (Figures 3A,B). PMCA expression in NEPs and NMPs were indiscernible from each other, both populations showing ubiquitous expression throughout the cultures for ATP2B1. In contrast to the RNAseq data, ATP2B2 was detected in both groups and it was mainly detected in dividing cells and also found in discrete sub-populations of cells, which SOX2 could not discriminate. In both groups ATP2B3 was ubiquitously expressed but at lower levels consistent with the RNAseq analysis. The ATP2B4 was strongly expressed in cell membranes of a sub-population of cells, however SOX2 expression could also not separate this population.

### PMCA expression in central and peripheral neural stem cells

**Figure 4:**
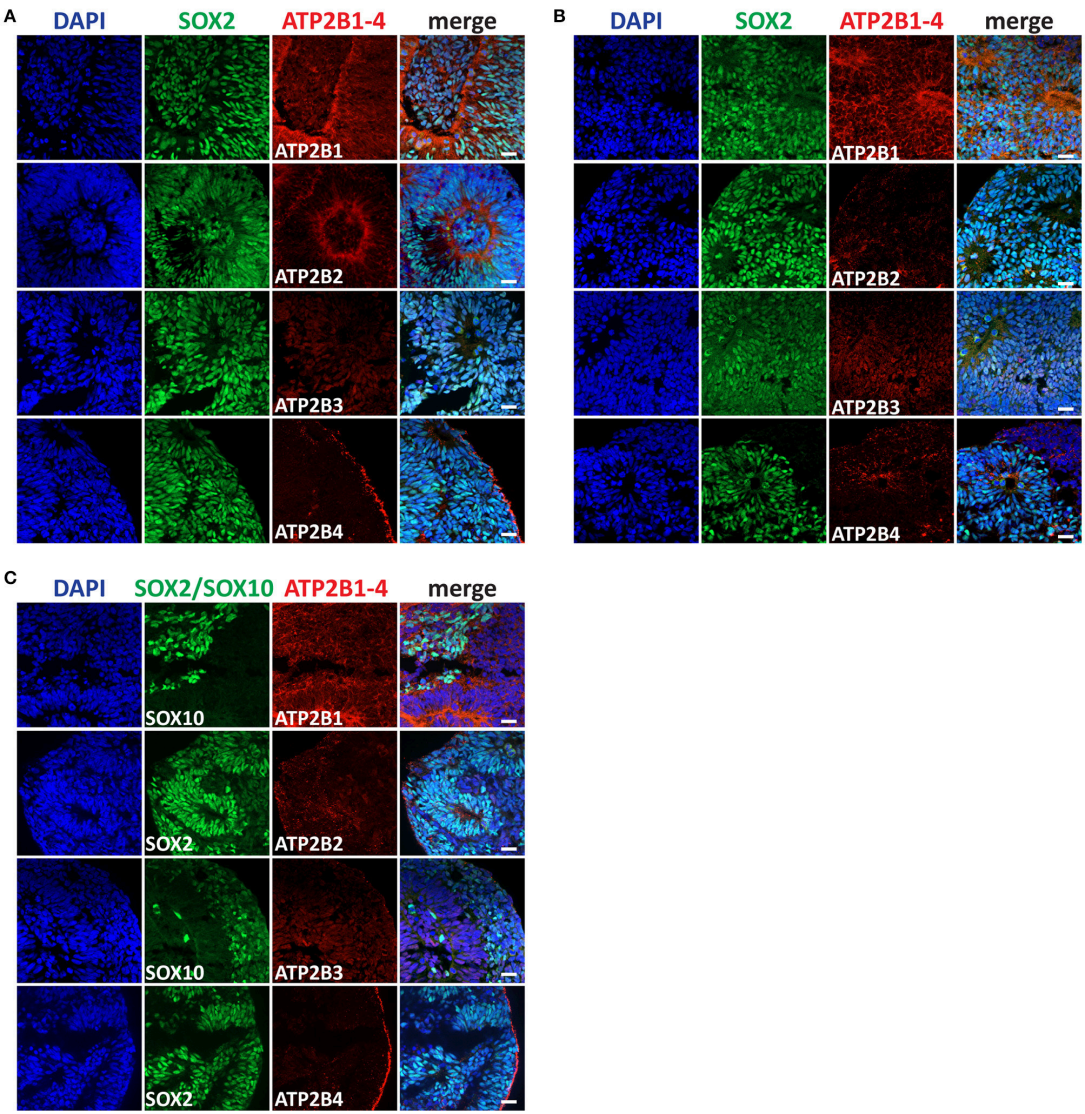
Immunocytochemistry analysis of ATP2B1-4 and SOX2 or SOX10 expression in **(A)** rostral neural stem cells (NSC), in **(B)** caudal NSC, and in **(C)** neural crest stem cells (NCSCs) on day 11. Scale bars = 20 μm.

We further characterized the PMCA expression at later differentiation states, the Rostral NSCs, Caudal NSCs and NCSCs (Figures 4A–C). In accordance with the RNAseq data and consistent with the early stem cell states all PMCA genes were detected within all groups. Interestingly in all three groups ATP2B1 expression was apically localized to the luminal regions of the neural rosettes, which also co-stained for SOX2. Furthermore, ATP2B2 within the Rostral NSCs was also apically localized to the luminal region of the rosettes and within the NCSCs ATP2B2 was selectively localized to the SOX2 positive cells. ATP2B4 expression was higher at the edges of the spheres except for the Caudal NSC groups, which showed a more ubiquitous expression.

### PMCA expression in mesencephalic dopaminergic neurons

**Figure 5:**
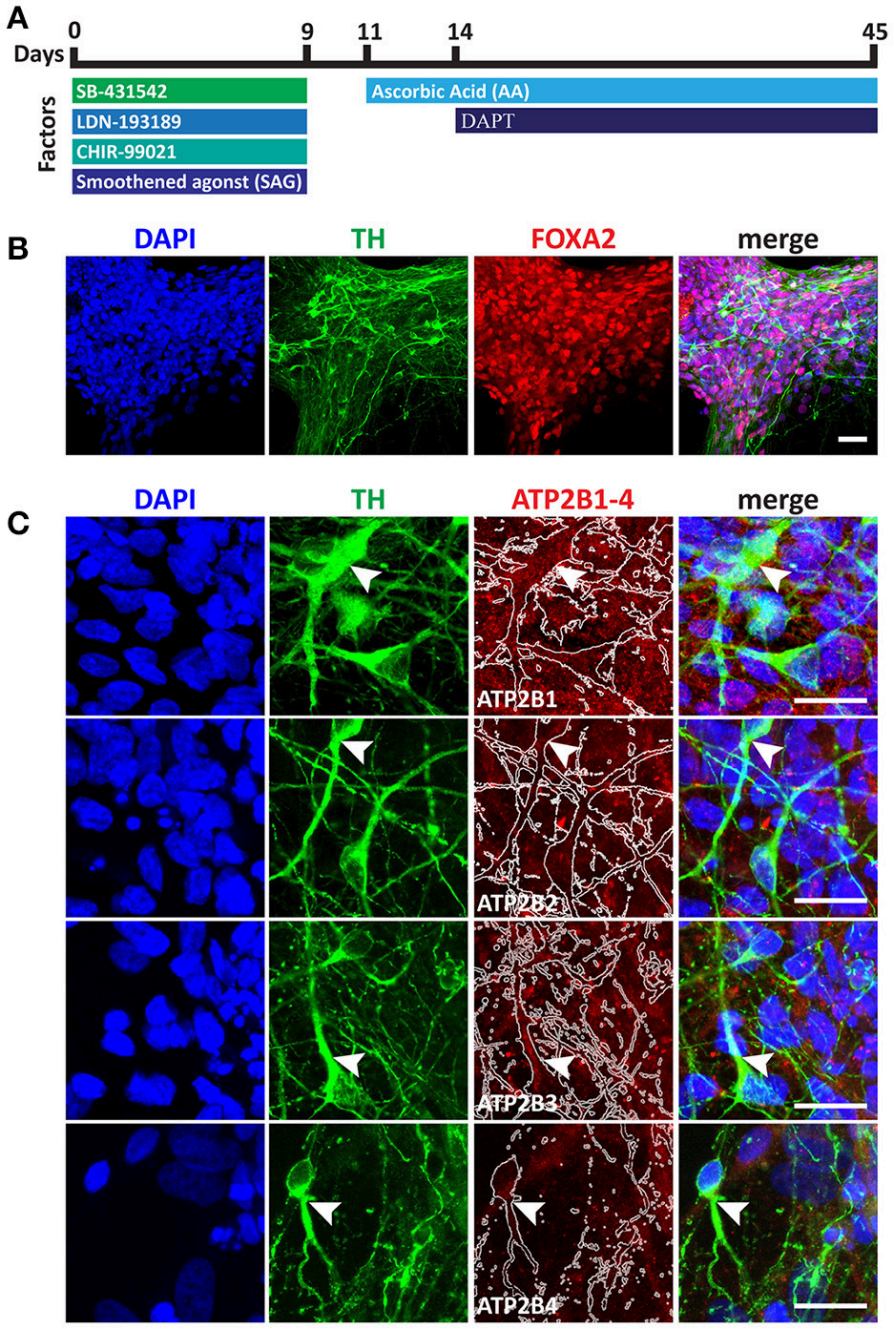
Immunocytochemistry analysis of ATP2B1-4 expression in mesencephalic dopaminergic neurons. **(A)** Overview protocol for making mesDA *in vitro* for 45 days. **(B)** Immunostaining of mesDA with double positive of FOXA2 and Tyrosine Hydroxylase (TH) markers indicates the efficient protocol. **(C)** Immunostaining of ATP2B1-4 and TH expression. Arrow heads point to the TH positive staining with ATP2B1-4 staining and white lines in ATP2B1-4 panels outlines TH positive staining. Scale bars = 20 μm.

Having identified the changing expression between the maturation states of the NSC we lastly sought to characterize the expression of PMCAs in mature neurons. We therefore investigated PMCA expression in mesDA neurons. mesDA neurons were differentiated *in vitro* for 45 days following our previously established protocol (Figure 5A). We first validated the protocol by staining differentiated cell populations with FOXA2 and Tyrosine Hydroxylase (TH), markers indicative of mesDA neurons, and identified neurons positive for both markers (Figure 5B). ATP2B1 was highly expressed and localized to the soma and the axons of the dopaminergic neurons. ATP2B2 and ATP2B3 were also expressed throughout the TH+ neurons, whilst ATP2B4 was detected weakly in the mesDA neurons (Figure 5).

## Discussion

Calcium acts as a major second messenger system within cells and as such it is involved in the regulation of numerous signaling pathways. During development stem cell fate is dictated by the precise temporal and spatial activation of signaling events, which requires specific ligand-receptor interactions and can rely on cytoplasmic calcium for transcriptional regulation (Lee et al., 2009; Thrasivoulou et al., 2013). This study has identified the expression changes of PMCAs that occur during early human stem cell states of the developing nervous system. In particular we have characterized the expression of PMCAs within: hPSCs, NEPs, NMPs, Rostral NSCs, Caudal NSCs, and NCSCs. Strikingly we observed a dynamic change in ATPase expression that coincided with the advanced differentiation states of NSC, which was independent of the differentiation of the NSCs (Figure 1C). We identified *ATP2B1* and *ATP2B4* as the most abundantly expressed, consistent with previous reports in other cell types and with ATP2B1 regarded as providing a housekeeping function for Ca^2+^ homeostasis (Okunade et al., 2004). Furthermore, *ATP2B1* expression significantly increased when differentiating from a pluripotent state to either a NMP or NEP, indicated a change in the transcriptional regulation of *ATP2B1* when transitioning to neural ectoderm. In contrast to *ATP2B1* and *ATP2B4*, the RPKM values of *ATP2B2* and *ATP2B3* were lower and *ATP2B2* was undetected in NMP. Despite the absence of detected transcripts, ATP2B2 proteins could be detected in NMPs, which was a sub-population of mainly dividing cells (Figure 1D). The depth of sequencing and the fact that ATP2B2 was only observed in a small subpopulation is the likely reason for the discrepancies between the sequencing and immunostaining results. Moreover, the differences between the RNAseq data and immunostaining results reflects the known divergence in transcript production and half-life to that of protein synthesis and turnover (Maier et al., 2009). Furthermore, our immunostaining also revealed a subcellular punctate localization for all the PMCAs, which suggests that the localization rather than the abundant of PMCAs are important for normal cellular function. The punctate localization of the PMCAs is likely formed to produce specific signaling complexes, which has been reported previously for ATP2B1-4, and the C-terminal tail of ATP2B4 has been shown to act as a signaling peptide involved in controlling its cellular localization (Marcos et al., 2009; Kenyon et al., 2010; Antalffy et al., 2013).

In contrast to mouse embryonic stem cells, which have been shown to only express *ATP2B1* and *ATP2B4*, we identified a transient pluripotent state with an atypical morphology that express ATP2B2 and still expressed POU5F1 and NANOG (Figure 2, Figure S2; Yanagida et al., 2004). These ATP2B2 expressing pluripotent stem cells may represent a primed state of hPSCs committed to differentiate but not yet having down-regulated all pluripotency genes (Kalkan et al., 2017; Liu et al., 2017; Smith, 2017).

Interestingly, in pluripotent cultures, ATP2B1 and ATP2B3 were highly expressed in cells undergoing mitosis. Both ionic current and PMCA activity have been shown to fluctuate during mitosis in the development of simpler eukaryotes (Zivkovic et al., 1991). Moreover, prevention of Ca^2+^ surges prior to or during mitosis result in inhibition of mitosis entry and exit, respectively (Steinhardt and Alderton, 1988). Thus, regulation of Ca^2+^ surges seems to be essential for cell division. Interestingly we could observe increased ATP2B1 and ATP2B3 in a period close to or during the anaphase-telophase transition, a stage which is dependent on a sustained increased Ca^2+^ level (Tombes and Borisy, 1989). Furthermore, nuclear Ca^2+^ can operate in an independent manner within the nucleus to that of the cytoplasm through the regulation of its own Ca^2+^ stores, with nuclear InsP_3_ being capable of regulating nuclear Ca^2+^ levels (Leite et al., 2003). In accordance with this, the cell cycle specific expression of PMCAs likely represents an altered requirement of cytoplasmic calcium regulation during nuclear envelope break down in cytokinesis (Stehno-Bittel et al., 1995).

Previous studies have identified ATP2B2 and ATP2B3 to be predominantly expressed in neuronal tissues (Stauffer et al., 1995; Strehler and Zacharias, 2001). Consistent with that we detected all PMCA isoforms in both NEPs and NMPs. Interestingly, at later stages in neural development we found ATP2B1 to be apically localized to the luminal regions of the neural rosettes. Active Ca^2+^ secretion to the lumen of the neural rosettes could be required for the generation of a specific lumen environment that promote neural tube closure (Abdul-Wajid et al., 2015; Suzuki et al., 2017). Furthermore, we identified ATP2B4 to be highly expressed at the edges of the neurospheres in the Rostral NSC and NCSC groups, however we could not identify a specific protein marker that could define this population. Nevertheless, an explanation for this could be that PMCA expression can be altered by changes in physical environmental rather than cell identity (Antalffy et al., 2013).

Dopamine release by mesDA neurons is dependent on precise Ca^2+^ fluctuations (Sgobio et al., 2014). We found that mesDA neurons expressed all four PMCAs. Alterations therefore in PMCA expression in mesDA neurons could contribute to mesDA neurons increased sensitivity to Ca^2+^ and hence to the PD pathogenesis. In support of this, impairment of Ca^2+^ homeostasis has been observed in iPSC-derived mesDA neurons from patients with heterozygous mutations for GBA1 (Schöndorf et al., 2014). These neurons showed an increase in basal Ca^2+^ levels and increased vulnerability to Ca^2+^ mediated endoplasmic reticulum (ER) stress. In addition, in PMCA overexpressing rats a neuroprotective function of PMCAs has been shown by its ability to decrease vulnerability to MPTP treatment (Brendel et al., 2014). Hence, strategies to regulate PMCA activity could serve as a neural protective mechanism in mesDA neurons by providing additional support for intracellular Ca^2+^ regulation.

## Conclusion

In summary, we have identified a dynamic change in the expression of P-type ATPases between distinct progenitor types. The transcriptional mechanisms that regulate these changes is still unknown and the functional significance of progenitor cell types requiring distinct P-type ATPase expression has yet to be elucidated, however it may reflect cell type specialization requirements. Most significantly we identified a novel expression timing of ATP2B1 and ATP2B3 during cell division of hPSCs and expression of ATP2B2 was observed in a subpopulation of hPSCs. Neural rosettes were identified as having a distinct apical localization of ATP2B1 to their luminal side. Lastly, we identified ATP2B1-4 were all expressed in mesDA neurons. Overall these results contribute to our understanding of the respective roles of the PMCA isoforms in neural development and their potential use as drug target for disease therapies.

## Author contributions

MD and PN: conceived the project and MD: designed the experiments; MC, SL, FF, CK, SB, JH, FX, and XL: performed the experiments; YL and LB: contributed RNA sequencing instrumentation and expertise in analysis; SL, MH, MC, and MD: analyzed data; MD, SL, and MC: wrote the manuscript; All authors reviewed the manuscript.

### Conflict of interest statement

The authors declare that the research was conducted in the absence of any commercial or financial relationships that could be construed as a potential conflict of interest.
