# Supplementary figures and images for: Central and Peripheral Nervous System Progenitors Derived from Human Pluripotent Stem Cells Reveal a Unique Temporal and Cell-Type Specific Expression of PMCAs
Source: Front Cell Dev Biol. 2018 Feb 6;6:5. doi: 10.3389/fcell.2018.00005 (PMC5808168; doi:10.3389/fcell.2018.00005)

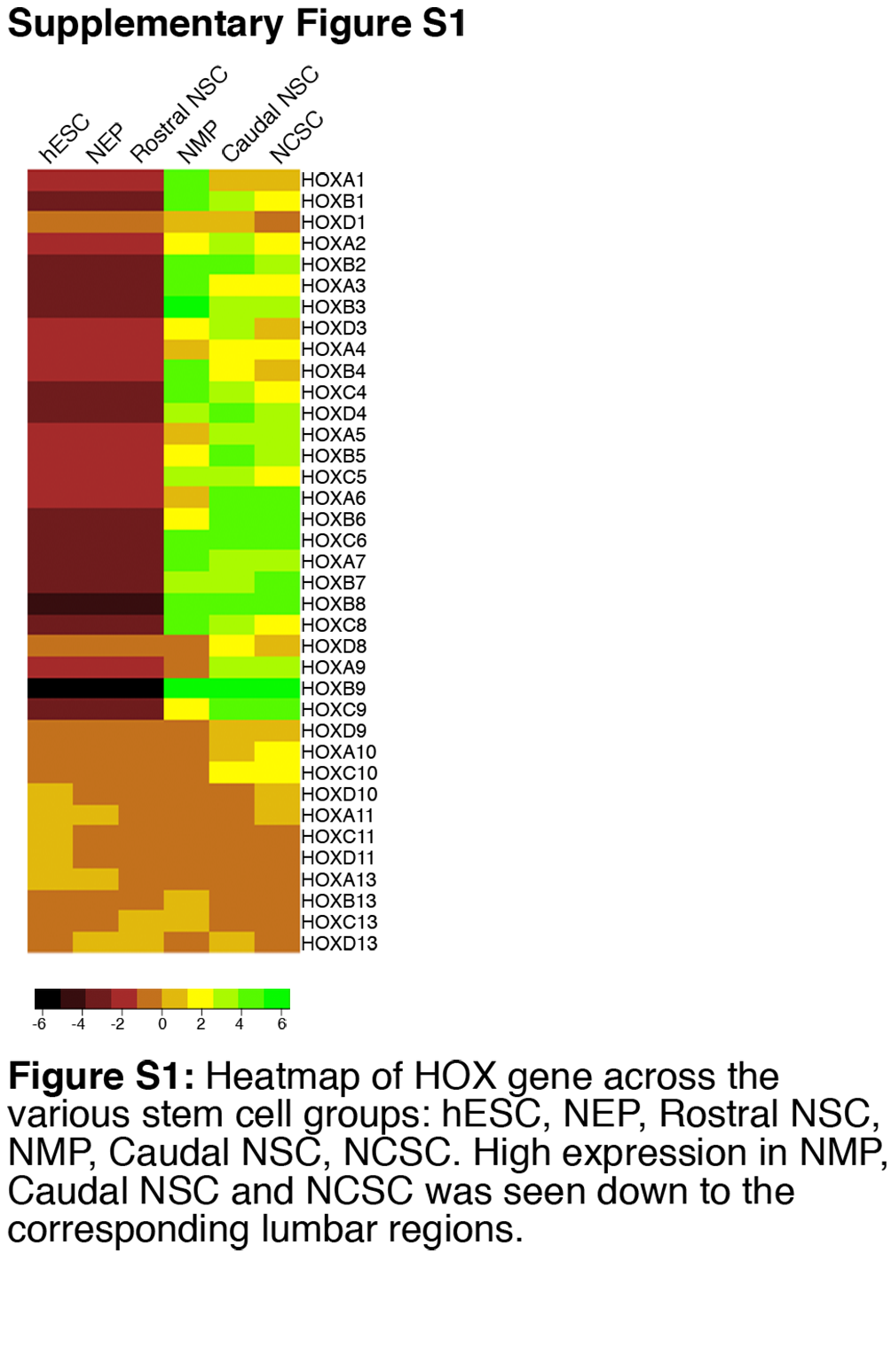

Supplement: Supplementary file 1 [file Image1.TIF]

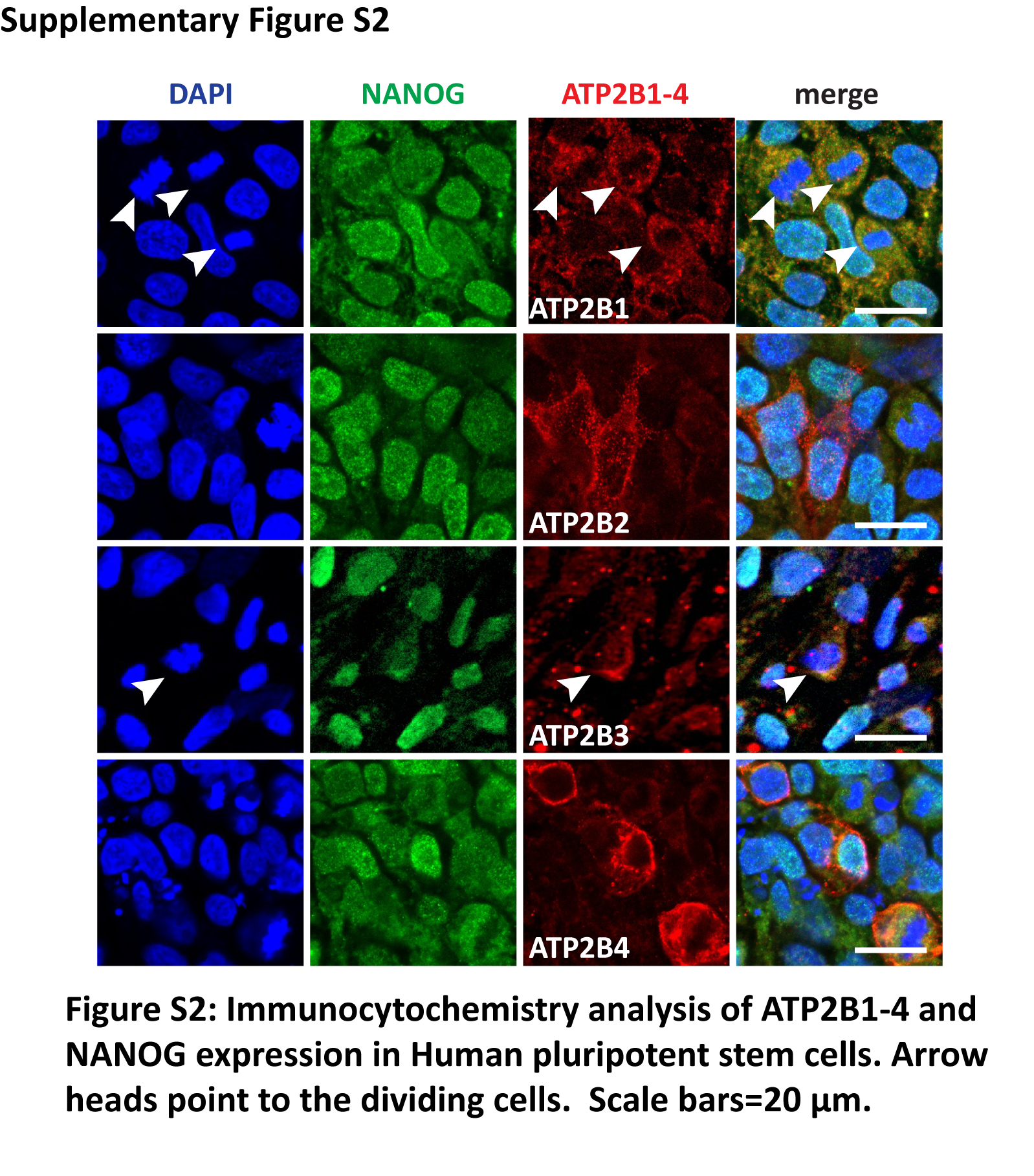

Supplement: Supplementary file 2 [file Image2.TIF]
